# Perioperative prevalence of deep vein thrombosis in patients with percutaneous kyphoplasty: A retrospective study with routine ultrasonography

**DOI:** 10.1097/MD.0000000000019402

**Published:** 2020-03-06

**Authors:** Wencan Fan, Tianzhu Qiao, Yongqing You, Jun Zhang, Jijian Gao

**Affiliations:** a Department of Orthopaedic Surgery, Daqing Oilfield General Hospital, Heilongjiang 163001; b Department of Nephrology, Affiliated Hospital of Nanjing Medical University, North District of Suzhou Municipal Hospital, Suzhou; c Department of Orthopedics, The First Affiliated Hospital of Soochow University, 188, Shizi Road, Suzhou 215006; d Department of Orthopaedic Surgery, Shengzhou People's Hospital, The First Affiliated Hospital of Zhejiang University Shengzhou Branch, Zhejiang, China.

**Keywords:** deep venous thrombosis, percutaneous kyphoplasty, pulmonary embolism, ultrasonography

## Abstract

In recent years, deep venous thrombosis (DVT) after spine surgery has received extensive attention, but perioperative prevalence of DVT in patients undergoing percutaneous kyphoplasty (PKP) is lacking.

To assess the perioperative prevalence of deep vein thrombosis (DVT) in patients undergoing PKP with routinely applied ultrasonography.

We reviewed 1113 consecutive patients undergoing PKP from January 2014 to August 2017. The surgical procedure was bilateral PKP. All patients were routinely examined with ultrasonography when admitted to the hospital and on the first post-operative day. Clinical signs of DVT were checked and recorded before examination.

Forty (3.6%) out of 1113 patients were diagnosed with DVT by ultrasonography. Of the 40 detected cases of DVT, only six (0.54%) patients presented with clinical signs of DVT, demonstrating that there were 34 (3.05%) asymptomatic cases. No patient presenting with clinically suspected pulmonary embolism (PE) was observed. Gender, body mass index (BMI), operative time, hypertension, diabetes, heart disease, and lower limb fracture were not significant risk factors for DVT (*P* > .05). In contrast, patient age, oncologic conditions, DVT history, and paraplegia appeared to be significant risk factors for DVT (*P* < .01). There was no significant difference in the incidence of DVT found between the three PKP surgical levels (*P* > .05).

The total incidence of perioperative DVT diagnosed with ultrasonography in patients undergoing PKP was 3.6%, of which only 0.54% was symptomatic cases. It is necessary to assess DVT using ultrasonography during the perioperative procedure of PKP, especially for high-risk patients.

Level of evidence: Level IV.

## 1 Introduction

Deep vein thrombosis (DVT) is viewed as a relatively rare complication in patients after spine surgery.^[1–3]^ Early diagnosis of DVT is important because of the risk of pulmonary embolism (PE) and potentially fatal sequelae. Reported incidence of DVT after spine surgery ranges from 0.3% to 31% in the literature in different patient populations.^[4]^ Alireza et al,^[5]^ in their single center report, found a cumulative symptomatic incidence of DVT as low as 0.83% after surgery and recommended dalteparin as prophylaxis. However, results from McClendon et al^[6]^ showed a high incidence of DVT (18.7%) with mechanical prophylaxis. In a recent study, the incidence of DVT was reported as high as 22.4% in patients undergoing lumbar Interbody fusion surgery, which may be related to advanced age, high-postoperative VAS scores, and blood transfusion.^[7]^ Several case reports feature an association between application of anticoagulation therapy and spontaneous spinal epidural hematoma (SEH),^[8,9]^ so the benefit of anticoagulation and the risk for SEH should be balanced. In view of the varied evidence regarding DVT in patients undergoing spine surgery, it makes clinicians hesitant of applying thromboprophylaxis.

DVT diagnosis has traditionally been performed using venography or ultrasonography. Ultrasonography is a convenient and noninvasive method for detecting DVT compared with venography. It also has proven safe and cost-effective, with a very high sensitivity and specificity (96% and 98%, respectively) for the diagnosis of DVT.^[10]^ Nowadays, minimally invasive surgery (MIS) is becoming a trend in orthopedic surgery.^[11,12]^ In a retrospective study, the total incidence of DVT after arthroscopic knee surgery was 14.9%.^[13]^ Fukushima et al^[14]^ demonstrated that the incidence of DVT was 6.94% during hip arthroscopy. In a randomized controlled trial (RCT), Xue et al^[15]^ found that the DVT incidence after proximal femoral nail anti-rotation (PFNA) surgery on treatment of intertrochanteric fractures was 3.3% (two out of 60). Based on these literature reports, the perioperative DVT cannot be ignored in MIS. Percutaneous kyphoplasty (PKP), one kind of minimally invasive spine surgery (MISS), can provide significant relief to patients with painful vertebral compression fractures (VCF) related to osteoporosis, multiple myeloma, hemangioma, or metastases.^[16–19]^ More than 50,976 patients reported through published articles have received the treatment of PKP in China.^[20]^

DVT incidence in PKP was presumed very low in many institutions and patients are normally underwent same-day procedures.^[21,22]^ To our knowledge, no report has described the perioperative prevalence of DVT in patients treated with PKP. The goal of this study was to determine the perioperative prevalence of DVT with ultrasonography in patients treated with PKP without antithrombotic or mechanical prophylaxis.

## 2 Materials and methods

### 2.1 Patients

This study retrospectively included 1113 patients admitted to Department of Orthopedics in our hospital. All patients underwent PKP, between January 2014 and August 2017. All patients received general anesthesia. The vertebral site lesions were located by C-arm and surgical methods were all used bilateral PKP. No thromboprophylaxis (e.g., mechanical squeezer, Plexi Pulse, or aspirin) were used during the perioperative period. Patients were discharged 2 to 3 days after surgery in average. The risk factors for DVT in these patients were also assessed. This study was approved by the ethics review committee of The First Affiliated Hospital of Soochow University, and written informed consent was obtained from all participants of the study.

### 2.2 Diagnosis of DVT

All patients underwent lower limb Doppler ultrasonography when admitted to the hospital and on the first post-operative day. The result was read by a physician and another two radiology specialists to get a clear diagnosis. If a vein or venous segment was not fully compressible, the results of ultrasonography were considered positive. Identified DVT was classified as proximal if it involved the iliac, superficial femoral or popliteal veins, with or without calf vein thrombosis, and as distal if it was isolated to the calf veins.^[23]^

### 2.3 Inclusion criteria

All patients over 18 years old treated with PKP were included. Patient gender, age, body mass index (BMI), hypertension, diabetes, oncologic conditions, DVT history, paraplegia, lower limb fracture, heart disease, operation time, PKP surgical levels, and distribution of DVT were recorded. Patients were excluded if they took anticoagulant such as aspirin, warfarin before hospital admission.

### 2.4 Data analysis

Statistical analyses were performed using IBM SPSS Statistics software, version 19 (SPSS, Chicago, IL). Results were expressed as the mean and the standard error of the mean (age, BMI, operative time). The results were compared between the two groups (DVT group vs non-DVT group). A two independent sample T test was used for comparisons of normally distributed data among the groups (age, BMI, operation time). Gender, hypertension, diabetes, oncologic conditions, DVT history, paraplegia, lower limb fracture, heart disease, and PKP surgical levels were statistically compared between the groups using the *χ*^2^ test. Statistical significance was defined as *P* < .01.

## 3 Results

Details of clinical and surgical data for PKP patients are shown in Table 1. During this 20-month period, 1113 patients (551 women) with a mean age (±standard deviation [SD]) of 58.3 ± 14.5 years treated with PKP. The mean duration (±SD) of the operation time was 68.5 ± 9.6 minutes. The mean BMI (±SD) was 24.4 ± 3.5 kg/m^2^. Nine hundred and forty-five patients underwent single stage PKP, 115 patients were received two-segment PKP and 53 patients were accepted multiple-segment PKP.

**Table 1:**
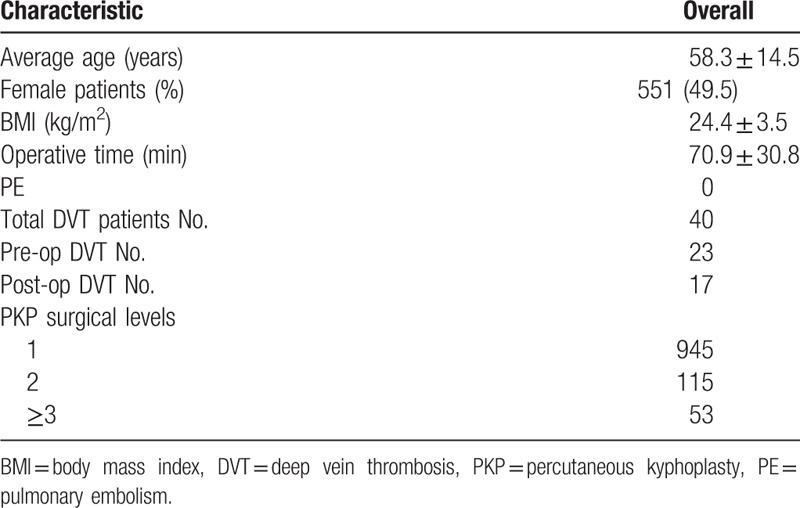
Clinical and surgical data for PKP patients.

A total of 40 (3.6%) patients were diagnosed with DVT with ultrasonography. Among them, 23 patients (2.1%) had DVT (proximal, six; distal, 17) before surgery. Seventeen patients (1.5%) developed new-onset DVT (proximal, six; distal, 11) after spine surgery. Of these 40 patients, only six (0.54%) had clinical symptoms of DVT, indicating 34 (3.05%) patients would have been missed without ultrasonography. No patient presenting with clinically suspected PE was observed. The distribution of DVT was shown in detail in Table 2. A total of 12 distributed in proximal veins and 28 in distal veins.

**Table 2:**
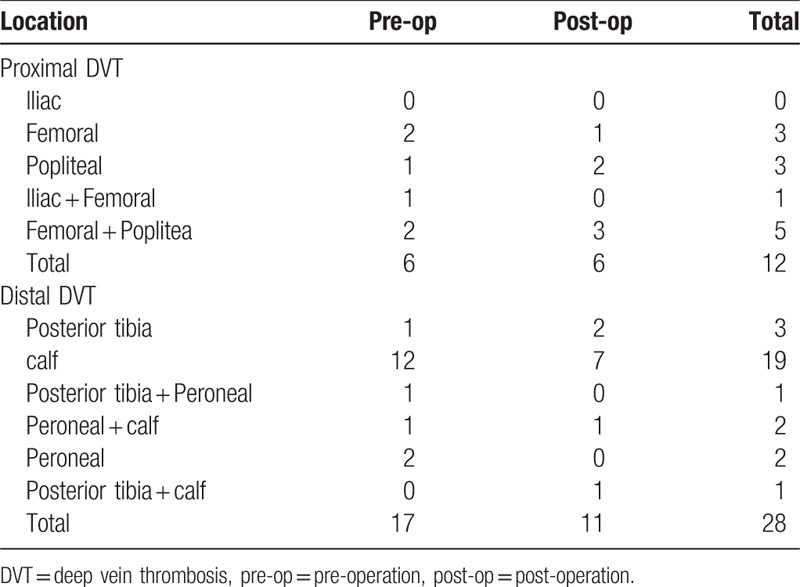
Distribution of identified deep vein thrombosis.

Association between clinical risk factors and DVT were analyzed in Table 3. There was no significant difference in the incidence of DVT found between the three PKP surgical levels (*P* > .05). Gender, BMI, operative time, hypertension, diabetes, heart disease, and lower limb fracture were not significant risk factors for DVT (*P* > .05). Age of the DVT group was 65.4 ± 10.6 years old, the non-DVT group was 58.1 ± 14.6 years old. A two independent sample *t* test showed that DVT group was significantly older than non-DVT group (*P* < .01). Cancer patients are at significantly increased risk for the development of DVT (17.5% vs 3.1%, *P* < .01). DVT history is an important high-risk factor for DVT (5.0% vs 0.19%, *P* < .01). The paraplegia patients in the DVT group were nearly 10 times higher than those in the non-DVT group (25% vs 2.7%, *P* < .01).

**Table 3:**
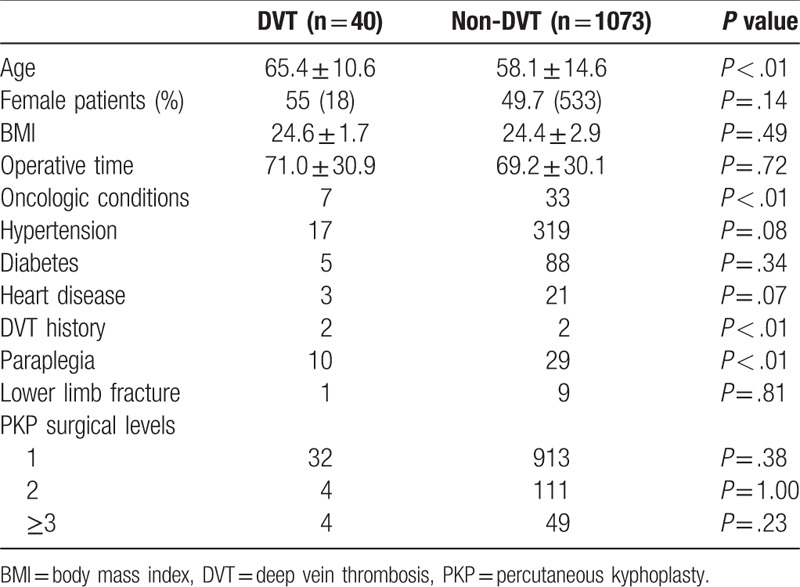
Association between clinical risk factors and deep vein thrombosis.

## 4 Discussion

A retrospective study of patients undergoing PKP was conducted in order to evaluate the incidence of DVT and the risk factors for perioperative DVT without antithrombotic or mechanical prophylaxis. So far, this study seems to be the first to report the perioperative prevalence of DVT in MISS patients treated with PKP in the literature. With ultrasonography, this study showed a 0.54% incidence of symptomatic DVT and a 3.05% incidence of silent DVT in 1113 patients undergoing PKP. In contrast, Akeda et al,^[1]^ detected that the incidence of perioperative venous thromboembolism (VTE) was 11% in spine surgery. MIS has the advantage of less trauma and shorter operation time. Namboothiri et al,^[24]^ found that the DVT incidence after spine surgery could be as low as 0.78% with no mechanical or chemical prophylaxis. However, ultrasonography was only performed in patients with symptomatic DVT. In addition, our incidence of silent DVT is 3.05%, almost six-fold that of symptomatic DVT. Without ultrasonography, nearly 85% (34 of 40) of the DVT patients will be missed and it could develop into a fatal PE. Besides, silent DVT was reported to increase the incidence of post thrombotic syndrome (PTS), its long-term clinical significance is still unclear.^[25]^ Therefore, the early diagnosis of asymptomatic DVT via ultrasonography should not be underestimated.

Age, oncologic conditions, DVT history, and paraplegia were shown to be significant risk factors for perioperative DVT in patients undergoing PKP in our study (*P* < .01). Age is a recognized risk factor for DVT, almost consistent with the other studies.^[26–28]^ This may be due to slow flow, hypercoagulability and the decreased in exercise with aging. Cancer patients increased risk for the development of DVT. In our study (17.5% vs 3.1%). Cancer patients are often deconditioned, neurologically impaired, and have chronic pain, which limiting their ability to ambulate, thereby increasing their risk for DVT during immobilization.^[29]^ In addition, DVT history is an important high risk factor for DVT (5.0% vs 0.19%). Stain et al,^[30]^ found a 2.6 (95% CI 1.2–5.9) times increased risk of recurrent VTE in patients with PTS, as compared to those without PTS, indicating patients with a history of DVT should be paid more attention to. In our study, the paraplegia patients in the DVT group were nearly 10 times higher than those in the non-DVT group (25% vs 2.7%). Paraplegia patients lost the motor function of the lower limbs, making the blood flow slow, rendering them at high risk of DVT.

The incidence of preoperative DVT is higher than that of postoperative DVT in our study (2.1% vs 1.5%). Song et al,^[31]^ detected that the incidence of preoperative DVT in patients, who are waiting for elective hip replacement for femoral neck fractures was (29.4%, 35 of 119). Coincidentally, Wakabayashi et al,^[32]^ in their retrospective study, found the prevalence of preoperative DVT in patients for total knee arthroplasty (TKA) was as high as 17.4% (56 of 322). In view of long duration of immobilization, preoperative DVT should not be neglected.

DVT especially proximal DVT is significantly associated with secondary PE.^[33]^ Furthermore, isolated distal DVT (e.g., calf muscle DVT) of the lower limbs can also be associated with subsequent proximal DVT and/or acute PE.^[34,35]^ In our study, ultrasonography scan was routinely performed from the iliac vein to the calf muscle vein, rather than only of the proximal vein. Indeed, nearly 70% (28 of 40) of the total DVTs were distributed at the distal side in our study. Therefore, the distal DVT should be normally scanned in clinical practice.

## 5 Limitations

Our study has several limitations. This study is a retrospective study, with a prospective study; we could come to a better conclusion with not being affected by too many risk factors for DVT. We only took CTA for the suspected patients for the diagnosis of PE. The incidence of symptomatic PE was historically low in our institution in PKP patients or for patients with any other spine surgeries so the asymptomatic PE patients may be missed. In addition, the incidence of this study was for patients during hospitalization. DVT may also occur in patients after discharge. Thus, the actual incidence of DVT after PKP could be higher than the findings in our study. At last, PKP patients in our institution were historically discharge 2 to 3 days after surgery. Furthermore, early exercise was encouraged, so DVT should be ruled out to prevent secondary PE on the first postoperative day with ultrasonography. For these reasons, we regret the lack of forward data.

## 6 Conclusions

The total incidence of perioperative DVT diagnosed with ultrasonography in patients treated with PKP was 3.6%, of which only 0.54% was symptomatic cases. It is necessary to assess DVT using ultrasonography during the perioperative procedure of PKP, especially for high-risk patients (advanced age, oncologic conditions, DVT history, and paraplegia).

## Author contributions

**Conceptualization:** Wencan Fan, Tianzhu Qiao.

**Data curation:** Wencan Fan, Tianzhu Qiao, Yongqing You, Jun Zhang.

**Formal analysis:** Wencan Fan, Tianzhu Qiao, Yongqing You, Jun Zhang.

**Investigation:** Jun Zhang.

**Methodology:** Tianzhu Qiao, Yongqing You.

**Validation:** Wencan Fan.

**Writing – original draft:** Wencan Fan, Tianzhu Qiao.

**Writing – review & editing:** Tianzhu Qiao, Yongqing You, Jijian Gao.
